# Challenges and Methods in Annotating Natural Speech for Neurolinguistic Research

**DOI:** 10.1162/nol.a.12

**Published:** 2025-09-05

**Authors:** Galit Agmon, Manuela Jaeger, Ella Magen, Danna Pinto, Yuval Perelmuter, Elana Zion Golumbic, Martin G. Bleichner

**Affiliations:** Department of English Literature and Linguistics, Bar-Ilan University, Ramat Gan, Israel; Gonda Multidisciplinary Brain Research Center, Bar-Ilan University, Ramat Gan, Israel; Frontotemporal Degeneration Center, Perelman School of Medicine, University of Pennsylvania, Philadelphia, PA, USA; Neurophysiology of Everyday Life Group, Department of Psychology, Carl von Ossietzky Universität Oldenburg, Oldenburg, Germany; Research Center for Neurosensory Science, Carl von Ossietzky Universität Oldenburg, Oldenburg, Germany

**Keywords:** annotation, computational linguistics, German, Hebrew, natural speech, temporal response function (TRF), transcription

## Abstract

Spoken language is central to human communication, influencing cognition, learning, and social interactions. Despite its spontaneous nature, characterized by disfluencies, fillers, self-corrections and irregular syntax, it effectively serves its communicative purpose. Understanding how the brain processes natural language offers valuable insights into the neurobiology of language. Recent neuroscience advancements allow us to study neural processes in response to ongoing speech, requiring detailed, time-locked descriptions of speech material to capture the nuances of spoken language. While there are many speech-to-text tools available, obtaining a time-locked true verbatim transcript, reflecting everything that was uttered, requires additional effort to achieve an accurate representation. We demonstrate the challenges involved in the process of obtaining time-resolved annotation of spontaneous speech, by presenting two semi-automatic pipelines, developed for German and Hebrew but adaptable to other languages. The outputs of these pipelines enable analyses of the neural representation and processing of key linguistic features. We discuss the methodological challenges and opportunities posed by current state-of-the-art pipelines, and advocate for new lines of natural language processing research aimed at advancing our understanding of how the brain processes everyday language.

## INTRODUCTION

### The Importance of Studying Spoken Language

Spoken language, the natural mode of human communication, plays a central role in cognition, learning and social interactions. During development, children acquire spoken language intuitively through exposure rather than formal instruction, revealing that the brain is wired to recognize and produce speech patterns (Hagoort & Indefrey, 2014; Kuhl, 2010). Moreover, natural spoken language, which predates written language, is extremely rich and reflects the creative and evolving nature of human expression, as evident in dialects, slang, the use of prosody and the interleaved presence of verbal and nonverbal elements (Levelt, 2000, 2001; Lieberman, 2002). Understanding how the brain encodes and processes natural speech can offer critical insights into the backbone of human communication, the ability to extract meaning from sound and the nature of semantic and contextual processes.

To date, the vast majority of research aimed at understanding speech processing in the brain has focused on a relatively narrow subset of speech features that are carefully designed and highly controlled for, in order to address specific research questions and hypotheses. These range from individual syllables (Jäncke et al., 2002; Obleser et al., 2006), through individual words (Chen et al., 2023; Humphries et al., 2006; ten Oever et al., 2022) or short sentences (Friederici et al., 2000; Humphries et al., 2006; ten Oever et al., 2022). Recently, researchers have been shifting to studying neural responses to continuous speech, in a less controlled fashion (Hamilton & Huth, 2020). These efforts have been afforded through the development of new signal-processing approaches, for functional magnetic resonance imaging (fMRI) and electro-/magnetoencephalography (EEG/MEG) and intracanial EEG/electrocorticography (ECoG) data (Crosse et al., 2016, 2021; Goldstein et al., 2022; Holtze et al., 2022; Iotzov & Parra, 2023; Jain et al., 2024; Kaufman & Zion Golumbic, 2023; Puschmann et al., 2024; Sueoka et al., 2024; Thiede et al., 2020), which offer new insights into higher-order aspects of speech processing in the brain, such as hierarchical processing of language, syntactic and semantic representations, hemispheric distinctions and mechanisms underlying selective attention to speech in noise (Brodbeck et al., 2018; Gillis et al., 2021; Inbar et al., 2020; Keitel et al., 2018).

However, even these studies typically use speech stimuli that do not represent the full richness of natural spoken language, using excerpts from audiobooks or other scripted materials—speech stimuli that are essentially spoken texts, are highly edited and deliberately constructed in terms of content and grammar and recorded by professional actors to optimize articulation, prosody and engagement (Blaauw, 1994; Face, 2003; Goldman-Eisler, 1968, 1972; Haselow, 2017; Huber, 2007; Mehta & Cutler, 1988; Wennerstrom, 2001). These speech stimuli differ substantially from the type of speech that people often produce and listen to in their everyday lives, which is spontaneous and dynamic, shaped by context and the interaction between speakers and their shared history and emotions (Shriberg, 2005; Tonetti Tübben & Landert, 2022). Unlike edited audiobooks, everyday speech is produced “on the fly,” it is often not highly polished in its structure and often contains disfluencies, pauses, self-corrections, ill-structured sentences and repetitions (Bortfeld et al., 2001; Clark & Wasow, 1998; Fox Tree, 1995; Wagner et al., 2015). Another common feature of natural speech is the presence of fillers, which are nonlexical utterances or filled pauses (e.g., “um,” “uh,” “you know”) that do not convey specific information but are nonetheless thought to be essential communicative cues (Arnold et al., 2004; Barr & Seyfeddinipur, 2010; Clark & Fox Tree, 2002; Corley et al., 2007; Corley & Stewart, 2008; Fox Tree, 2001; Fraundorf & Watson, 2011; Watanabe et al., 2008) and disambiguating syntactic structures (Bailey & Ferreira, 2003; Tonetti Tübben & Landert, 2022). This results in everyday speech being substantially more complex and less well structured than scripted speech, characterized also by the personal style of the speaker and the specific nature of the interaction.

In this article, we spotlight the efforts to annotate real-life speech in a time-resolved fashion. Specifically, we ask whether current annotation approaches that have been developed for rehearsed speech are suitable when studying real-life speech. We present two pipelines as case studies of the native languages of the authors, German and Hebrew. We explicitly outline the steps involved in these speech analysis pipelines—steps that are often taken for granted and insufficiently documented. Through these examples, we highlight and discuss the challenges and dilemmas involved in annotating naturalistic speech stimuli and the strengths and weaknesses of currently available automated tools.

Importantly, although we focus on two specific pipelines for two specific languages, the principles discussed here can be extended to any language, particularly non-English languages. While many speech analysis/annotation tools seem robust when applied to English, they do not necessarily generalize to languages with different phonetic or orthographic systems. Therefore, another goal of this article is to spotlight not only the challenges of analyzing real-life speech, but also how these challenges become potentially even more pronounced in non-English languages.

### Annotating Natural Speech for a Time-Resolved Analysis

Recent methodological advances now allow studying how the brain processes speech in real time, moving beyond isolated elements to analyzing language as it is naturally delivered—continuously. One such approach is the use of temporal response functions (TRFs; Crosse et al., 2016, 2021; Lalor & Foxe, 2010; Simon, 2015), an analytic approach for linking features of continuous speech with features of neural activity recorded while listening to the speech, primarily using data from electrophysiological recordings with high temporal resolution such as EEG, MEG, and ECoG (Crosse et al., 2016, 2021; Lalor & Foxe, 2010; Simon, 2015) but can also be applied to fMRI recordings with low temporal sampling (Hausfeld et al., 2024; Puschmann et al., 2017). Common to approaches that aim to analyze neural responses to continuous speech is that they involve aligning vectors that represent a particular feature of the speech with vectors capturing the ongoing neural response, and comparing between them (e.g., by performing a linear reverse-correlation or other mathematical operations). In the case of applying TRF analysis to neural recordings using EEG/MEG, for example, this alignment must be accurate down to the millisecond level.

A key feature of these approaches is that they are not limited to a particular feature of speech, but are flexible. One can choose any feature of speech to analyze—acoustic, phonetic, lexical, semantic, or syntactic—either in isolation or in combination. Indeed, many different speech features have been used in such analyses, ranging from acoustic features, such as the speech envelope, onsets, or spectral profile, to linguistic properties, including phoneme identity, lexical status, cloze probability, or semantic probability (Agmon et al., 2023; Brodbeck et al., 2018, 2022; Gillis et al., 2021, 2022).

However, in order to apply these methods, speech features must be systematically extracted, in a precise and time-resolved manner, ideally automatically). Some features, such as the acoustic envelope, can be derived easily through well-defined algorithms. However, features capturing linguistic information are more complex and require specific choices or assumptions. For example, determining the probability of each word involves identifying word boundaries in the acoustic signal, linking speech acoustics to lexical elements, and using a relevant language corpus or large language model (LLM) to assign values to each word (Heilbron et al., 2022; Stanojević et al., 2023). The accuracy of this process depends on the quality and reliability of available tools for analyzing audio, speech, and language, and the methodological choices made along the way can significantly impact the final output.

Common to all time-resolved analyses are three key requirements: (1) determining which features to include in the model, depending on the specific hypotheses to be tested; (2) ascribing numerical or categorical values to elements in the speech stimulus to reflect the manifestation of each feature; and (3) identifying the precise timing of each element in the speech stimulus and creating vectors representing the time course of each speech feature, which are perfectly aligned with the neural signal.

A well-structured and replicable annotation of the speech input provides the foundation for selecting features, ensuring transparency about which aspects of the speech signal are incorporated into the analysis and which are disregarded.

### Approaches to Transcription: Clean Verbatim Versus Full Verbatim

All current approaches to speech annotation require its transcription—that is, transforming the audio recording to a text-based representation of what was uttered. Nowadays, many automatic speech-to-text tools are readily available, designed to do just that. However, as we discuss throughout, many tools have been optimized for ideal speech, such as audiobooks and movies, and the dynamic nature and variability of natural speech present unique challenges to this process. In particular, determining what constitutes an adequate transcript of a particular speech recording, and what level of accuracy and details is required can depend on the specific application and goals of the transcription.

*Clean verbatim* transcription focuses on readability and clarity, omitting unnecessary fillers, stutters, and false starts, unless they convey crucial information. Clean verbatim transcription enhances the flow of the written content, ensures that the essence and meaning of the spoken word is preserved without the clutter of nonsemantic verbal habits, and is often viewed as ethically appropriate, avoiding potential misrepresentation of the speaker’s intent or competence. This type of transcription is frequently used for transcribing interviews, creating subtitles in media production, and in most commonplace speech-to-text algorithms (e.g., Eftekhari, 2024; McMullin, 2023).

However, some contexts require capturing a *full verbatim* transcript. This type of transcription is meticulously detailed, including nonlinguistic utterances from the speakers, such as coughs, stutters, and even ambient noises. Such comprehensive detail ensures that the transcript is maximally informative, serving as an exact record that might be necessary, for example, in legal contexts or for evidence scrutiny.

In our research, we prioritize full verbatim transcription because it is the most accurate representation of what a person actually heard, and therefore of what their brain encodes and processes. This approach also allows us to capture the richness and complexity of natural speech, with all its disfluencies and imperfections, as a feature rather than a bug. Only with this detailed and precise level of transcription can we gain a comprehensive understanding of the elements that drive neural responses and underlie speech processing in the brain. Using a full verbatim transcription provides the basis for the next step in our methodology: a rich set of features that captures the complexity of spontaneous speech across multiple levels of analysis.

### Aim for Rich Feature Sets

A rich set of features to choose from provides a comprehensive description that would ultimately lead to a fuller understanding of how speech is processed by the brain. While accurate full verbatim transcription is necessary for this, it is not sufficient. Researchers need to decide which features to extract from the transcribed speech, and how to represent them in vector-form. This can lead to difficult choices, since speech can be described at different levels and along many dimensions. For example, a specific utterance can be described according to its acoustic envelope or onsets, its lexical features (e.g., predictability, part-of-speech [POS] category), or even its grammatical function. Moreover, an utterance can be described through its prosody or through additional nonverbal elements that convey its intended pragmatics (e.g., rate, followed by a pause, followed by a sigh). And then there is the matter of the irregular, dysfluent, and sometimes incorrect nature of spontaneous speech, characteristics which ideally should also be captured as features of the speech (Agmon et al., 2023; Christodoulides & Avanzi, 2015).

It is not our intention here to delineate which are the “correct” features to use in a given research project. However, in discussing our annotation pipeline we highlight the considerations and challenges that go into transforming segments of natural speech into an array of time-resolved vectors that adequately represent the richness and complexity of this stimulus and can be used to advance our understanding of speech processing.

## PIPELINE FOR ANNOTATING NATURAL SPEECH

**Figure 1.**
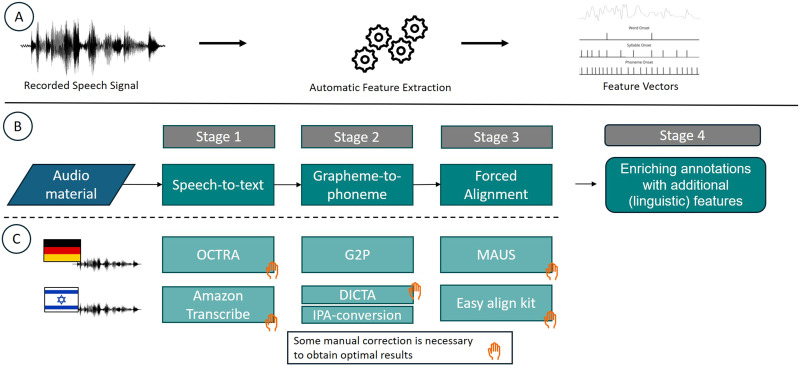
Speech annotation pipeline. (A) Based on the acoustic signal an automatic feature extraction leads to time-resolved feature vectors. (B) starting with an audio recording of some speech, a speech-to-text tool is used to convert the spoken language into written text. In grapheme-to-phoneme conversion the written text (graphemes) is transformed into their corresponding sounds (phonemes); this phonetic transcript is then temporally aligned with the audio file to identify the exact time onset of words or phonemes. This stage is essential for all the following stages. (C) The pipeline we have used for German and Hebrew material, with an indication on the level of automation.

Here we describe a pipeline for annotating continuous speech stimuli, and specifically natural speech. As described in Figure 1, the pipeline consists of the following main stages:

**Stage 1—Speech-to-text conversion**: In this stage, the speech is transcribed into written text, providing a textual representation of the audio signal.
**Stage 2—Grapheme-to-phoneme conversion**: This stage determines which phonemes correspond to each word in the text, creating a phonetic representation of the speech.
**Stage 3—Forced temporal alignment and hierarchical parsing**: This stage precisely determines the time points when each phoneme occurs in the audio signal and, as a result, accurately identifies the onsets and offsets of syllables and words.

Stages 1–3 result in time-resolved vectors that describe the temporal boundaries and lexical/phonetic representation of all speech-elements in the stimulus. They form the basis for Stage 4.

**Stage 4—Enriching annotations with additional (linguistic) features**: This stage is where researchers can choose to describe the speech elements (phonemes/syllables/words/other) according to specific features of interest, such as lexicality, POS, probability, role in sentence, and syntactic correctness.

Ideally, this pipeline (at least Stages 1–3) should be as automatic as possible, so it can be applied to large bodies of speech materials. Here, we make use of several available automated tools for German and Hebrew speech (Figure 1C) used to convert speech to text, transform text to its phonological representation, and extract the exact time points of specific phonemes. We also highlight the strengths and weaknesses of specific procedures and address some language-specific challenges that can arise.

### Speech Material

The annotations pipeline was developed and tested using recordings of German and Hebrew speech material.

#### German speech material

Natural speech stimuli were recordings of unscripted monologues in German. Six people (3 female / 2 male / 1 diver) were instructed to tell stories about various topics of their everyday life (i.e., hobbies, subjects of study, holidays, part-time job). In total, the stimulus-set consists of over 300 minutes of material containing separate monologues between 3 and 20 minutes and is available on Zenodo (Daeglau et al., 2023).

#### Hebrew speech material

Natural speech stimuli were segments from a publicly available podcast in Hebrew. The materials were spoken in the same male voice and covered a variety of topics that are of interest to the public (e.g., history, science, culture). The stimulus set consists of 54 minutes of speech. Although these materials were somewhat scripted, they are delivered in an informal manner and contain fillers and other conversational elements.

### Stage 1: Speech-to-Text

#### Overview

The first stage in the annotation process is converting the spoken speech to text. This is necessary since the tools and procedures used in all subsequent stages depend on having a text-based representation of the speech. Nowadays, automated, easy-to-use, speech-to-text tools exist for most languages, including on large commercial web-based platforms (such as Amazon Web Services (Leeper, 2020), Google (Google Cloud, n.d.), or Microsoft (Microsoft Azure, n.d.). These tools accept audio-files as input and return a text-file with its transcription, in the source-language orthography. Some of these services also provide coarse time stamps indicating the timing of each word within the audio file. These tools are generally tailored to produce linguistically correct output (clean verbatim) that is suitable for, for example, voice commands and often include processing steps that detect and remove unnecessary repetitions or fillers.

#### Language-specific approach

German speech-to-text transcription was done using the BAS web service Orthographic Transcription (OCTRA; Draxler & Pömp, 2017) and we selected Google as the provider for automatic speech recognition (ASR). We chose this provider after comparing its performance on a subset of speech materials relative to the other providers (IBM and Fraunhofer). Hebrew speech-to-text transcription was done using the Amazon Transcribe tool (Leeper, 2020). We chose this provider after comparing its performance on a subset of speech materials relative to the other providers (Google, Microsoft, Whisper, and Vonage).

#### Manual corrections

In both languages, the ASR transcribed text file was generally of high quality but required manual corrections to ensure that the text faithfully represented the actual spoken narrative. The performance of ASR systems is usually assessed using the word error rate (WER), a metric that describes the percentage of errors in the total word count. Under ideal conditions today’s ASR systems can achieve WER rates as low as 5%, and WER rates between 5% and 10% are considered high-quality. In our speech samples, approximately 10%–30% of words were not transcribed correctly. Ironically, most of the required corrections were due to the fact that ASR algorithms are trained to fix alleged errors in spoken language. These include: repetitions, shortened versions of words (e.g., [in German] “ne,” “nen” instead of “ein,” “eine,” “einen”; [in Hebrew] “t’shma” instead of “tishma”), mispronunciations, fusions of two words (e.g., [in German] “fürn” instead of “für ein”; [in Hebrew] “z’tomert” instead of “zot omert”), and words that differ in their colloquial speech form from their standard written form (e.g., [in German] first-person verbs are often spoken without the last letter “e”, e.g., “hab,” “mach”) while in the transcript the standard language form was introduced (e.g., “habe,” “mache”); [in Hebrew] “lisoa” instead of “linsoa”). In addition, verbal fillers (e.g., “ähm,” ”err,” “umm”) as well as nonverbal sounds (e.g., coughs) are automatically removed in the transcript.

While this cleaning-up can be advantageous for many speech-to-text applications (e.g., to create subtitles for a movie, transcribe a lecture or give voice-based commands), for our purposes this was a disadvantage since our goal is to model the neural response to the actual speech stimulus heard by listeners, for which we need a full verbatim transcript.

We also note a few specific word-categories that require special treatment and manual corrections. One category is numbers, which are not dealt with well by automatic speech-to-text transcribers and are sometimes written out as digits or omitted entirely. For our annotation process, numbers need to be written out in full (e.g., nineteen ninety three). Similarly, abbreviations commonly appear in the transcript in their short form, rather than in their full form as uttered (e.g., “km” instead of “kilometer”). Another category that automatic transcription tools have difficulty with are loanwords (e.g., use of terms in English within Hebrew/German speech; e.g., “atmosphere,” “instrumentals,” “beats”), slang words (e.g., “Histalbet” [in Hebrew]), rare proper nouns or names (e.g., “Tillysee” [in German]).

Since the forced alignment stage (Stage 3) requires a transcription that accurately represents the speech phonetics, all of these errors required manually correcting the transcription to match the actual phonemes and sounds present in the audio file.

#### Accurate time-stamping

Although the tools used here can provide time stamps for each word, when comparing these to the audio itself, we found that these were not sufficiently accurate (with latency shifts of tens to hundreds of milliseconds). Therefore, we disregarded these time stamps and instead performed our own time-stamping using forced alignment algorithms (Stage 3). For successful forced-alignment, the transcription not only needs to accurately represent the speech phonetics, but also needs to be presented in an orthography that can be “read” by the forced aligner. This requires a grapheme-to-phoneme transformation of the text, a process in which written text (graphemes) is transformed into the corresponding spoken forms (phonemes).

### Stage 2: Grapheme-to-Phoneme

#### Overview

The grapheme-to-phoneme process is highly language dependent and generally assumes standard pronunciation. Therefore, the specific challenges are also language dependent. Using our language case studies, we give a few examples below.

#### Language-specific approach: German

Grapheme-to-phoneme conversion for the German transcriptions was carried out using the BAS webservice G2P (Reichel, 2012; Reichel & Kisler, 2014), a web application for converting orthographic text into a canonical phonological transcript corresponding to a standard pronunciation. G2P reads the transcript and estimates the most likely phoneme sequence that a standard speaker is expected to articulate, in German. It uses statistically trained decision trees, POS tagging, and morphological segmentation to improve the decision process. Additionally, G2P is trained on a large set of pronunciations to build a language-specific pronunciation dictionary. As output, we chose to represent the speech using the language-independent and machine-readable phoneme symbol inventory Speech Assessment Methods Phonetic Alphabet (X-Sampa; Wells, 1995). The output of the web application is a text file containing for each word the standard pronunciation in X-Sampa. This processing step was fully automated.

#### Language-specific approach: Hebrew

Grapheme-to-phoneme conversion for the Hebrew transcriptions required two stages: (1) adding vowels to the Hebrew transcript; (2) transforming the text from Hebrew orthography to the International Phonetic Alphabet (IPA).

##### Adding vowels.

Hebrew orthography consists of mostly consonants, with the vowels either inferred or added as diacritic marks. This can result in ambiguities in the text representation that are not originally present in the phonetic representation. To ensure that the text correctly represents the speech phonetics, we used an automatic tool (Shmidman et al., 2020) to reinstate diacritic marking of vowels above and below the Hebrew letters in the transcribed text. This stage was mostly automatic, but in some cases required manual corrections, when the automatic tool assigned vowels that did not correspond to the speech audio.

##### IPA conversion.

Since most forced alignment algorithms do not read Hebrew letters, we converted the text into the IPA (Ladefoged & Halle, 1988). The IPA representation overcomes the use of different orthographic representations across languages and is an accurate text-based representation of the speech phonology (including vowels, distinguishing between homographs, and consolidating across homophones). We used the online tool Zemereshet that automatically converts Hebrew text with vowels to IPA (Cohen, 2019).

#### Challenges and considerations

Grapheme-to-phoneme conversion is typically based on standard pronunciations. However, for some words colloquial pronunciation differs across speakers, affected dialect, slang, or cultural context. Deviation from the standard pronunciation can lead to errors in grapheme-to-phoneme conversion, and/or in use of phonemic representation that do not accurately reflect the audio. An example from German is the word “zwei” (two), which is often pronounced as “zwo” to avoid confusion with “drei” (three) in noisy environments. The speech-to-text tool might correctly identify “zwei” based on context, leading the grapheme-to-phoneme conversion to use the standard pronunciation “tsvai,” even though the speaker actually said “tsvo.” Improving grapheme-to-phoneme conversion accuracy would require training these models in a more culture/dialect-specific manner or incorporating the audio input itself into the process.

### Stage 3: Forced Temporal Alignment and Hierarchical Parsing

#### Overview

Forced alignment refers to the process of aligning phonological transcripts with their corresponding sound files to achieve precise, time-resolved annotations. The outcome from this process is a transcript where each linguistic unit is accurately given a time stamp reflecting its onset and offset position in the audio file. The resolution or level of description of forced alignment can vary, and to a large degree depends on the user’s needs. Here we sought to obtain time stamps for three levels of linguistic units: individual phonemes, syllables and words.

It is important to acknowledge that automatic tools for forced alignment, even if they work on non-English language, are still generally language-specific, in the sense that they are trained on data from a particular language. Therefore, applying these tools blindly on languages other than English may produce unpredictable results (Chodroff et al., 2025) as demonstrated below for Hebrew.

#### Language-specific approach: German

Forced alignment of the German speech was performed using the Munich Automatic Segmentation webservice MAUS (Kisler et al., 2017; Schiel, 1999), which uses an HMM (Hidden Markov Models) based Viterbi Decoder to find the best alignment between speech signal and pronunciation model. In detail, it uses the phonological transcript, identifies the segment in the audio-file that corresponds best to each phonetic unit and provides time stamps for its onset and offset. It also labels each segment to reflect its phonetic representation in X-Sampa, the phonological representation of the word in X-Sampa, as well as the orthographic representation of the word. It is important to note that the phonetic and phonological representation may differ, if the speaker deviates from the standard pronunciation derived by the grapheme-to-phoneme conversion. MAUS may detect this deviation and segment and label the speech according to what was actually spoken.

**Figure 2.**
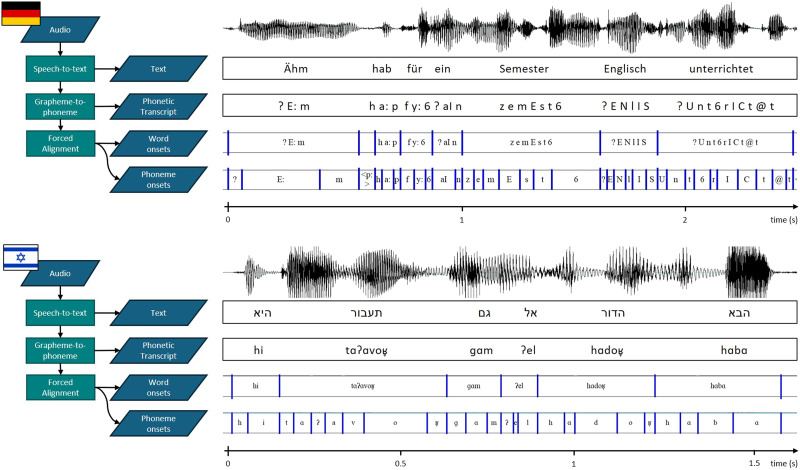
Illustration of the German (upper part) and Hebrew (lower part) speech annotation process and corresponding output examples. Starting with a recording of speech, a speech-to-text tool is used to convert the spoken language into written text. In the grapheme-to-phoneme conversion the written text (graphemes) is transformed into their corresponding sounds (phonemes), and this phonetic transcript is then temporally aligned with the audio file to identify the exact time onset of words and phonemes.

Additionally, the MAUS algorithm can detect interword silence intervals, which we defined as any pause longer than 10 ms. The output frame rate of the MAUS algorithm was set to 1 ms, to optimize the temporal resolution of the boundaries of each linguistic unit. Figure 2 illustrates the upper part the German speech annotation pipeline and the corresponding output of a short example.

#### Language-specific approach: Hebrew

Forced alignment of the Hebrew speech was performed using Praat software (Boersma, 2001) and the Hebrew version of the EasyAlign (EasyAlignIPA), developed by the Open Lab for Media and Information (OMI Lab; Silber-Varod et al., 2023). EasyAlign is a Praat-based forced alignment tool based on HMM which is based on ASR (Goldman, 2011). The process identifies the section of an audio file that best fits a given unit of text (provided in IPA format).

The EasyAlign segmentation process is made up of three major steps: macro-segmentation, phonetization, and phone segmentation. During the macro-segmentation step, short utterances of the speech (corresponding roughly to clauses; segmented manually) are aligned in time with the portion of the auditory stimuli containing this utterance. Then, in the phonetization step all the phonemes in the utterance, which are represented as IPA symbols, are converted to SAMPA using a Hebrew phonetic lexicon table (Silber-Varod et al., 2022) that contains each of the Hebrew phonemes and the average duration it takes to pronounce them. Last, in the phone segmentation step each utterance is parsed into three levels, represented as separate tiers in Praat: words, syllables, and phonemes. For the phoneme tier, vowels and consonants are parsed as separate units (example: Y | a | Ɂ | e | l). For the syllables tier, the onsets and offsets of syllables are identified and parsed based on the location of vowels. For the words tier, boundaries between words are identified based on spaces in the transcribed text. Then the forced alignment procedure is applied separately for each level, to precisely align the audio to each unit, across tiers. The final output of this pipeline (Figure 2) is a Praat TextGrid file, detailing the identity and time stamp of each speech element within the audio file, with separate tiers for each level of representation, including the utterances.

While our pipeline relies on language-specific tools—MAUS for German and EasyAlignIPA for Hebrew—some modern aligners, such as the Montreal Forced Aligner, offer pretrained models for a wide range of languages. These tools represent valuable steps toward reducing the Anglocentrism of speech technology. However, their performance on spontaneous, disfluent speech and less-resourced languages remains variable and often requires manual verification (Chodroff et al., 2025; Fromont et al., 2023).

#### Manual adjustments and corrections

Both forced alignment algorithms (MAUS used in German and EasyAlignIPA used in Hebrew) work best when given short segments of speech. Since we worked with speech materials that were relatively long (>30 s), forced alignment was performed in a two-tiered manner. First, the full speech transcription was manually separated into segments corresponding roughly to single sentences, which were entered into the forced aligner to identify the section of the audio file corresponding to each segment (macro-alignment). This produced a rather coarse indication of the onset and offset of each segment. We then applied the forced alignment a second time, focusing only on a particular segment of the speech, to identify the time stamps of more specific and local linguistic features—words, syllables, and phonemes (micro-alignment).

Although this automatic process yielded relatively good time-stamping, manual corrections were still required to ensure that onset and offset were correctly marked. These were performed in Praat, based on both the auditory input and visualization of the speech waveform.

We found that the onset timing was generally more precise than offset timing. This is likely due to the continuous nature of speech, which does not contain clear pauses between words/syllables/phonemes and therefore the offset of one can be identical to the onset of a subsequent word. For instance, when a word ended with a vowel and the next word started with a vowel, the boundary between them was misaligned (e.g., [in German] “konnte ich” or “schaffe ich”; [in Hebrew] “lo yoda’at”).

Similar to Stages 1 and 2, here too pronunciation was not always accurately represented in the transcription. Therefore, words that were drawn out, mumbled, or slurred sometimes resulted in forced alignment errors. Other elements that required manual corrections were the beginning of silent periods and non-speech sounds such as breathing and coughing, which are sometimes erroneously treated by the forced aligner as speech. Based on our experience, this manual correction phase typically requires between 5 and 20 minutes of work per 1 minute of speech, depending on factors such as recording quality, speech clarity, and the precision needed for downstream neural analyses. This heuristic may serve as a practical benchmark for researchers when planning annotation projects.

In general, the quality of the forced alignment is highly dependent on the quality of the recording, the level of background noise, and the clarity of speech pronunciation, and of course on the pronunciation and acoustic models used to train these algorithms. For time-resolved analyses of neural data, precise temporal alignment of sensory stimuli is essential to yield meaningful results (Woodman, 2010). Temporal imprecisions in stimulus onset information can be detrimental to analysis of averaged evoked potentials (Hairston, 2012). Carta et al. (2023) investigated the effect of temporal imprecision on TRF analysis by introducing temporal jitter into the speech envelope. They found that temporal jitter up to 25 ms leads to only slight reductions in TRF amplitude and prediction accuracy, whereas larger jitter between 25 and 50 ms caused degradation of responses. In our speech samples we analyzed the temporal jitter between the uncorrected and manually corrected version of the forced alignment to identify a potential impact on time-resolved analyses. We found that approximately 10% of the word and 4% of the phoneme onsets exceeded a temporal jitter of 20 ms after manual correction, indicating that the forced alignment algorithm works reasonably well given an accurate speech-to-text and grapheme-to-phoneme conversion. Based on Carta et al. (2023) our results suggest that, given accurate speech-to-text and grapheme-to-phoneme conversions, manual correction of the forced alignment may not substantially impact TRF outcomes. We currently lack neural data to quantify how such jitter in discrete linguistic units affects TRF analyses. However, we are in the process of collecting such data, which we anticipate will warrant a separate publication. For now, a pragmatic heuristic may be to aim for <20 ms jitter in corrected annotations, especially when aligning high-level linguistic features to neural data, but this guideline awaits empirical validation.

### Stage 4: Enriching Annotations With High-Level Information

Up to this point, we described the process and challenges of obtaining a time-resolved description of the basic units of speech (onset and offset of phonemes, syllables, and words; Stages 1–3). This level of annotation can be useful by itself to investigate many research questions, addressing the representation of different units in the brain and dissociating their acoustic versus lexical representations (Di Liberto et al., 2015; Hertrich et al., 2012; Lesenfants et al., 2019).

However, many research questions regarding speech processing go beyond simple demarcation of hierarchical units but pertain to the identity and meaning of the words themselves, their linguistic characteristics and their discourse role within a spoken utterance. Examples include questions about the predictability of a word within a given context (Brennan & Hale, 2019; Goldstein et al., 2022), the effect of surprise or uncertainty (Brodbeck et al., 2018; Frank & Willems, 2017), differences between content versus function words (Agmon et al., 2023; Bell et al., 2009), concrete versus abstract words (Binder et al., 2005; Roxbury et al., 2014), opening versus closing words (Agmon et al., 2023), incremental syntactic parsing (Brennan et al., 2016; Nelson et al., 2017; Stanojević et al., 2023), and composition of meaning over the course of a sentence (Bruera et al., 2023; Desbordes et al., 2023). To address these types of research questions, annotations are required that go beyond detection of word/syllable/phoneme boundaries, but ascribe each unit (usually words) a value that represents the specific feature that is of interest in a particular study.

However, how to best determine these values on a word-per-word basis for natural speech, and what tools are available to do so optimally, is still an open question. Here, we discuss several types of higher-level annotations that are of particular interest in the context of neural processing of natural speech—pertaining both to linguistic features and nonverbal characteristics of speech—and highlight current challenges for obtaining reliable annotations.

Natural language processing (NLP) tools have been gaining dominance over manual annotation of linguistic features. These include automatic parsers (e.g., SpaCy; Honnibal & Johnson, 2015), the Stanford parser (Chen & Manning, 2014), and the Charniak-Johnson parser (Charniak & Johnson, 2005), which are trained to provide the syntactic structure of the input, and large language models (LLMs) that are trained on next-word prediction (e.g., BERT, GPT, Llama). Although these NLP tools can be extremely useful in many domains, applying them to transcripts of speech, and to natural speech in particular, can be tricky. This is because the training of these tools involves primarily written text, and therefore their performance depends on assumptions about the material that do not necessarily hold for speech. In our own attempts to use automatic parsers or LLMs to create linguistic annotations for natural speech, we encountered four main challenges: the necessity to segment speech into sentences, non-traditional syntax, the presence of disfluencies, and nonverbal communicative features.

#### Spoken language lacks clear sentence markers

A fundamental assumption when training and using automatic parsers and LLMs is that the input is composed of well-formed full sentences. In text, boundaries between sentences are represented using punctuation (periods, commas, and question and exclamation marks). Parsers are trained on annotated examples that are segmented based on the textual punctuation. LLMs learn the role of these symbols in delineating boundaries between sentences and then use them when analyzing new textual materials. Without punctuation, essential information for accurate analysis of the sentence is missing. In fact, LLMs and parsers can give erroneous or unpredictable results if given text without punctuation marks. Attempting to parse a transcript with no punctuation enforces a syntactic structure of a single complete sentence, resulting in ascribing wrong POS roles (Westpfahl & Schmidt, 2013) or parsing errors (Agmon et al., 2024). For example, without punctuation, a parser would analyze the utterance “I like you. Help me.” as if “you help me” is a subordinate clause (as in “I like that you would help me”). As for LLMs, word predictions are influenced by the surrounding context, so the presence or absence of punctuation can lead to different predictions. This holds true even when the overall syntactic structure remains largely the same between the punctuated and unpunctuated versions of the text. For example, when using an LLM to predict which word should replace [XXX] in the sentence “three blind mice [XXX] how they run,” a model would generate different predictions depending on the presence of a comma separating the two clauses. With a comma included, distill-BERT (Sanh et al., 2019) predicts the top three tokens as “see,” “describing,” and “showing.” In contrast, when the comma is omitted, the model predicts “:”, “describe,” and “discover” as the three most likely tokens. Since punctuation is often a stylistic choice, its impact on the output of NLP tools presents a challenge when analyzing speech.

Therefore, in order to use NLP tools for linguistic feature extraction, it is critical to provide them with accurately punctuated textual inputs. However, spoken language does not contain overt lexical markings of punctuation, and listeners infer sentence boundaries using a combination of syntactic analysis and prosodic elements such as pauses, speech rate, and intonational contours. Moreover, punctuation can also be a matter of culture, language, or stylistic preference (e.g., Baron, 2001; Steinhauer & Friederici, 2001), and different styles may result in inconsistent analyses. The full verbatim transcript of speech, derived in Stage 1 of our pipeline using commonly available ASR tools, does not contain punctuation markings. Although there are some automatic tools that can perform sentence segmentation on text (e.g., spaCy; Honnibal & Johnson, 2015, and wtp; Minixhofer et al., 2023), these are not always accurate for transcription of speech, since they are based only on textual input with no access to prosodic cues. In order to accurately punctuate transcriptions of natural speech, automatic tools would need to be able to combine textual analysis and prosodic information, as listeners do naturally. Multimodal language models (e.g., Radford et al., 2022) are trained to transcribe speech and insert punctuation that can then be used for defining sentences. However, current performance on natural speech seems yet suboptimal (Agmon et al., 2024). Alternatively, punctuation could be re-inserted manually, based on a native listener’s evaluation. However, besides being an extremely tedious and inefficient process, manual punctuation can be subjective, as mentioned, and it can also suffer from inherent ambiguities, stemming from the nature of natural speech, as discussed in our next point.

#### Spoken language does not conform to traditional linguistic structures

The nature of natural speech makes it inherently difficult to demarcate the boundaries between sentences, be it manually or using automatic tools (Shriberg, 2005). This is because natural speech often includes fragments, false starts, and corrections, making it challenging to decide what constitutes a *sentence*. The fluid and nonlinear nature of spoken language can blur the lines between sentences. This raises questions about the relevance and application of traditional sentence concepts in the context of speech.

Take, for example, the following hypothetical speech segment: “The girl is telling her | what do you call it | I know this word | nanny | that she doesn’t want to go to sleep yet” (the symbol “|” is used to mark positions in the sentence that should contain a pause). Here, the main message/clause (“the girl is telling her nanny that she doesn’t want to go to sleep yet”) is interrupted by the speaker’s thoughts (“what do you call it,” “I know this word”), which are not embedded in the main clause like a dependent clause would be. It is very difficult to apply traditional sentence segmentation or syntactic analysis to this type of input, and yet listeners have no problem understanding the intent of the speaker and the hierarchical clause-structure, despite its irregularity. Other examples are sentences that are incomplete or contain repetitions and self-corrections, such as: “I was part of a group on Facebook, I mean WhatsApp, where we … every day someone sends, you know, a poem or a … sometimes more than one, could be a picture too.”

Trying to describe these types of utterances within traditional linguistic structures, raises a broader question of whether the concept of a sentence, as defined in the traditional linguistic view, is even applicable to natural spoken language. Grammatical rules describe planned language, which adheres more easily to predictable structures. These rules may not effectively capture the structure and dynamics of spoken language, where disfluencies and spontaneous circumlocutions disrupt complete structure. It is possible that, for the purpose of linguistic annotation of spoken language, we may need to develop new categories of linguistic units that better describe the type of utterances that humans naturally compose when speaking “on the fly” (Haselow, 2017; Pietrandrea et al., 2014).

#### Spoken language contains disfluencies

Even if we could agree on the “correct” way to parse natural speech, despite its irregularities and incomplete nature, these features remain problematic for linguistic feature extraction using NLP tools. This is because these models are trained on language that usually does not contain superfluous insertions, and therefore they do not deal well with disfluencies such as fillers, self-corrections and false starts (Devlin et al., 2019). Importantly, a full verbatim transcription *does* include disfluencies as tokens. This creates a high mismatch between the probability space of the test set and the statistics of the speech input. Importantly, this extends to other types of tokens, not just disfluencies—the probability space of the test set includes probabilities for punctuation, emojis, and other symbols that do not exist in speech. It is therefore not surprising that when a speech transcription is fed into an LLM for prediction, results can be drastically different depending on whether it is a clean transcription or a full verbatim transcription.

Since disfluencies are hardly represented in textual input, they are highly unpredicted by LLMs. It is hard to determine whether cognitively, the brain is surprised every time a disfluency is detected, but disfluencies may serve as an attentional-orienting cue (Fox Tree, 2001; Fraundorf & Watson, 2011). However, it is clear that for properly studying the neural effects of disfluencies, they have to have a significant representation in the training set, which is currently not the case. The underrepresentation of disfluencies in the training set may affect not just the probability scores of disfluent tokens, but the probability scores of the non-disfluent tokens (real words) as well. The probability scores of words is determined by their context, and that context containing disfluencies may have an effect, as evident by the following anecdotal example: For example, using the bidirectional model BERT (Devlin et al., 2019), the average probability of the random made-up Hebrew sentence “I don’t always think that I am right in everything that I say” is 0.24. Augmenting the sentence with disfluencies (“**I umm** I don’t always think that I am **like** right in everything that I say **uhh**”), the average probability on the real words (not including the augmented disfluencies) is 0.12. A detailed assessment of the severity of this issue on real data would require a tailored modeling approach to examine how disfluencies influence probability estimates—an interesting direction for future research that goes beyond the scope of this manuscript.

The presence of disfluencies affects not only probability scores, but every aspect of the linguistic analysis, including the syntactic tree of a sentence. By definition, disfluencies do not have a syntactic role and are not semantically integrated into the final meaning of the sentence (Agmon et al., 2024). However, a parser that receives an input with disfluencies will provide an output of a syntactic structure, with these disfluencies receiving a syntactic position in the tree, sometimes resulting in very twisted structures. Recently, there have been some attempts to automatically detect disfluencies, but these are not yet commonly used (Christodoulides & Avanzi, 2015; Kulick et al., 2023). Incorporating these approaches more broadly in automatic tools, perhaps by providing users with the option of working with clean or full verbatim transcriptions, would be extremely beneficial for improving research-tools pertaining to natural speech processing.

### Annotation of Nonverbal Features

Besides annotating speech according to linguistic features, much of the critical information for speech processing is not necessarily conveyed through the words themselves. These include contextual gestures, intonation, and body language, which do not appear at all in the textual representation of the stimulus. However, these features are fundamentally important for listeners to understand spontaneous speech, and as discussed above, often compensate for the lack of a “correct” linguistic structure. Some prosody cues or acoustic features, such as the use of irony, surprise, sarcasm, and word stress, can change the meaning of a sentence completely (Bryant & Fox Tree, 2002; Cheang & Pell, 2008; e.g., the sentences “oh, what lovely weather” and “thanks a lot” can be said in earnest or sarcastically). Similarly, the use of head nods (Al Moubayed & Beskow, 2009), hand gestures (Obermeier et al., 2015; ter Bekke et al., 2024), and eye movements (Al Moubayed & Beskow, 2009; DePaulo et al., 2003) can facilitate the comprehension of the verbal message or charge it with new meanings. Since these additional cues are absent from the text-based transcription of the utterance, NLP tools that are trained on complete well-formed text-only sentences are unlikely to interpret and analyze them in the same way a human listener would.

Prosody plays a fundamental role in speech processing, not only by conveying speaker intent, emphasis, and discourse structure, but also by marking phrase boundaries. As real-life speech lacks punctuation, it is prosody that serves as a crucial cue for accurate parsing (Hawthorne & Gerken, 2014; Langus et al., 2012; Lehiste, 1973; Lehiste et al., 1976; Price et al., 1991; Shattuck-Hufnagel & Turk, 1996). It is therefore essential to incorporate annotations of prosodic features that serve as cues for sentence segmentation and help resolve syntactic ambiguities. Among the prosodic features that support syntactic parsing, word duration and pauses are particularly influential, though other features, such as pitch declination and vocal fry, may also contribute to distinguishing between different syntactic structures and clarifying ambiguous sentences. By integrating prosodic information, automatic speech processing tools can more effectively model the natural rhythm and structure of spoken language, leading to more accurate syntactic interpretation.

Ideally, a complete description of a given speech stimulus would include a systematic way to annotate and describe relevant speech features, in a time-resolved manner. This would allow integrating linguistic and nonverbal attributes in the same model, affording unique insight into their complementary roles in speech processing. Some basic tools to this effect do exist, such as the ToBi system for annotating distinctive prosodic events (Jun, 2014; Rosenberg, 2010), which could be used to enhance the full verbatim transcription with prosodic cues. In addition, some tools exist for automatic extraction of prosodic contours from audio files (Mahar et al., 2021; Suni et al., 2017), and analysis of hand and head-gestures from videos (Akakin & Sankur, 2011; Alon et al., 2005). However, clearly this domain requires more effort, development, and resources, in order to create a set of tools that could provide comprehensive descriptions of the nonverbal features of natural speech and streamline their analysis at scale.

## DISCUSSION

In this article, we present the challenges and some blind spots in applying standard annotation procedures to spoken language, and in particular to spontaneous speech. This annotation process is critical for an accurate time-resolved analysis of neural responses to speech, but it can also be useful for many other research purposes aimed at understanding behavioral and neural aspects of speech processing.

Ideally, this process would be fully automated, leveraging the remarkable advances in NLP algorithms over the past decade. However, critically, spontaneous speech drastically diverges from the data used as training sets for these tools. ASR and NLP models are trained on a clean textual version of language, which does not align with the features observed in the full verbatim transcription of speech. Critically, a full verbatim representation of the speech is essential, both for methodological and scientific reasons, since only a full verbatim representation accurately captures what the brain actually hears and responds to. For this reason, we seek to preserve all aspects of the linguistic content in the verbatim transcription, including filler words, hesitations, and nonverbal vocalizations. The challenges we highlighted—such as difficulties aligning non-speech sounds and filler words, limitations in automation, and the abstraction of sound into text before time alignment—are not just technical obstacles but directly impact the validity of our neurophysiological analyses.

As neuroscience researchers, we approach these annotation methods as end users, relying on them to work effectively for our purposes rather than developing them ourselves. Our primary concern is their applicability to studying neural signals at the millisecond level across a wide range of processing hierarchies. Therefore, we require tools that are both highly temporally precise and faithful to the actual uttered content—a need that does not always align with the objectives of automatic speech processing for two main reasons. First, it is difficult to generate a full verbatim transcript using these tools, as they are designed to correct disfluencies and imperfections. Second, it is difficult to perform automatic linguistic analysis on full verbatim transcripts of natural speech, since these are generally trained on idealized text-based corpora that usually consist of full, grammatically correct and punctuated sentences. Therefore, although automatic tools can assist in various stages of the annotation process, manual verification and correction is usually necessary, which sometimes entail making difficult decisions when interpreting ambiguous or context-dependent elements of speech (e.g., boundaries between words, what constitutes a sentence). Since natural speech deviates from “ideal” language in many ways, it may be necessary to create new descriptors of linguistic elements of speech and syntactic structures that are better suited for capturing the complex and irregular nature of these materials.

The issues raised here become even more severe when considering that most human speech is produced in conversation with others, adding levels of complexity pertaining to conversational dynamics. Annotating that type of speech would likely require an additional set of discourse features, including interruptions, overlap, turn-yielding, back-channeling, sentence completion, echoing, reformulation, and more. While discussing this type of speech at length is beyond the scope of the current manuscript, the current work lays the foundation for thinking about how one might go about annotating conversational speech as well.

Turning to a broader discussion, we would like to highlight the current obstacles and set the vision in progressing toward using realistic natural speech in neurolinguistic research.

### Going Through Text

Many of the problems we encountered in the process of obtaining a time-resolved annotation of speech material from an audio file are related to the fact that we are forced to go through text. To recap, our process begins with a speech-to-text conversion, followed by a grapheme-to-phoneme conversion, used to perform temporal alignment to obtain time stamps of phoneme and word onsets. Each of these stages can introduce multiple sources of error, as detailed in the sections above. Prominent problems include the automatic “cleaning up” of mispronunciations, hesitations, and fillers by automatic speech-to-text tools, which then need to be re-inserted into the transcription, as well as erroneous grapheme-to-phoneme mapping due to variations in pronunciation across speakers and contexts. Moreover, many of the challenges regarding more advanced annotations, such as analyzing linguistic features and the inability to take into account prosodic and nonverbal cues, stem from the fact that current approaches rely on using a textual representation of the speech.

**Figure 3.**
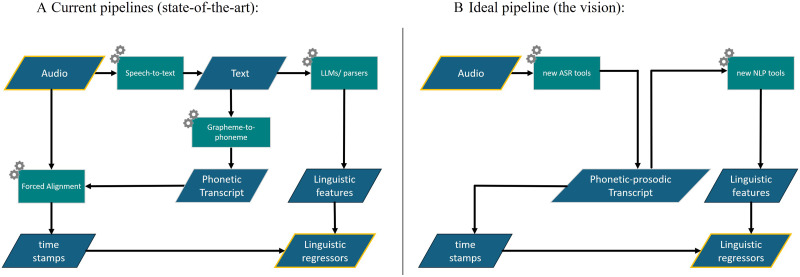
Current pipelines versus ideal pipeline. (A) A diagram showing the current process of obtaining a time-resolved feature representation from an audio file, going through text. Audio material is transcribed using speech-to-text algorithms, which is used for two purposes: to extract linguistic features of the text (e.g., part of speech tagging) and to guide the process of phoneme identification and temporal forced alignment. (B) An alternative process that does not go through text, but rather features are derived directly from the audio. This process is more optimal for processing speech, but it requires the development of new ASR tools and new NLP tools that are trained on speech, along with a representation of speech which is not dependent on specific orthographies (e.g., international phonetic alphabet) and incorporates prosodic cues that are important for parsing in the absence of punctuation.

As an alternative to going through text, we put forth the idea that a full verbatim transcription and annotation may be obtained more reliably with tools that use only the audio input (see Figure 3). This includes an ASR tool that will reliably represent the phonemes in the input and their timings, as well as NLP tools that are trained on such data rather than on orthography. Surprisingly, to the best of our knowledge, there is no tool available that can provide direct audio-to-phonetic annotation of speech, without going through textual representation.

Current NLP tools such as parsers or LLMs are trained on text which is given in the particular orthography of the language. However, if ASR and NLP tools were developed and trained directly on speech material, represented phonetically, allowing for variation in pronunciation and dialect and representing prosodic aspects of the utterance, it may provide solutions to many of the ambiguities and challenges encountered using the current pipeline.

In line with our advocated message, audio-based spoken language models can offer a promising direction towards an ideal pipeline. These models take into account the acoustic signal in addition or instead of textual input (Arora et al., 2025). Their potential extends beyond accurate transcription to include the annotation of paralinguistic features such as speaker identity, emotion, and prosodic cues like pitch, making them strong candidates for nuanced, context-sensitive analysis of spontaneous speech (Arora et al., 2025).

Phoneme-level transcription directly from audio can already be achieved using wav2vec 2.0 (Baevski et al., 2020), a self-supervised speech representation model developed by Meta. It is pretrained on raw audio, learning meaningful representations from the speech signal alone, without textual transcripts. During fine-tuning, wav2vec 2.0 can be adapted to map speech to phonemes, words, or other linguistic labels, and it could, in principle, be further fine-tuned for linguistic annotation tasks (Lakhotia et al., 2021). Another self-supervised audio-based model is HuBERT, also developed by Meta (Hsu et al., 2021). Like wav2vec 2.0, HuBERT generates dense vector representations from audio at high temporal resolution, though it relies on clustering of pseudo-labels as part of its pretraining objective. These frame-level representations, analogous to characters in text, could theoretically serve as units for tokenization and transformer-based language modeling, enabling a purely acoustic model that captures the distributional properties of spoken language without relying on orthography. Notably, the audio used in pretraining for both models primarily consists of clean, read speech (e.g., LibriSpeech or Libri-Light; Kahn et al., 2020; Panayotov et al., 2015). Spontaneous speech is underrepresented in most large-scale ASR corpora used for pretraining, limiting models’ exposure to real-life speech during pretraining. In any event, to the best of our knowledge, although such an orthography-free approach has been generally proposed (Lakhotia et al., 2021), developing speech models that operate without reliance on orthographic representations is still only in the experimental stage.

### Are Text-Based Features Suitable for Annotating Speech?

A broader issue, which we touched upon briefly with regard to Stage 4, is whether the linguistic concepts that work well for annotating written text are even suitable for natural speech.

Take, for example, the extensive use of probabilities in describing per-word features, which have traditionally been obtained using cloze probability or *n*-grams (Brown et al., 1988; Heilbron et al., 2022; Shaoul et al., 2014), and more recently by training LLMs on large databases (Devlin et al., 2019). Since these probabilities are extracted from large corpora of text (e.g., newspapers, Wikipedia), they most likely do not faithfully represent the probability of words occurring in spoken language, which, arguably, includes different discourse-levels and sentence structures and relies immensely on nonverbal prosodic and pragmatic cues (Shriberg, 2005). Therefore, alongside the technical problems that arise when trying to apply text-based tools to transcripts of natural speech, we must also ask ourselves more broadly: Are the text-based features that these models are trained on the most relevant and correct ones for describing speech?

Linguistic categories such as POS tagging, and syntactic parsing have clear rules in every language. However, for natural spoken language, it is not always straightforward to determine. The crux lies in the fact that in natural speech, the speaker cannot go back and edit parts of the utterance that reflect the messy process of constructing the message. When producing language on the fly, in an unplanned manner, the hearer is exposed to the hesitations, corrections, and loss of track of the speaker. These dynamics result in linguistic structures that do not necessarily adhere to the standard grammar rules of the language yet are still perceived as natural and acceptable (or at least understandable) by the hearer. Chomsky describes this phenomenon as performance, as opposed to competence of language which refers to the knowledge of grammar, the language of an ideal speaker-hearer who is unaffected by cognitive limitations that could result in performance errors (Chomsky, 1965). According to this perspective, performance errors are not part of the grammar system and therefore are not governed by the same set of rules that govern the processing of an ideal input. As such, it is likely that the brain processes performance errors differently from “true” grammatical errors, and that the brain overcomes performance issues on its way to ideal abstract representation (Agmon et al., 2023). Conversely, usage-based linguistics does not distinguish between competence and performance and argues that there is no ideal abstract representation that is extracted from the messy input (Bybee, 2006; Tomasello, 2005). As such, the linguistic analysis that was developed to describe ideal planned language is irrelevant for describing speech, and a new framework with more relevant annotations should be developed. The debate between generative linguistics and usage-based linguistics is still ongoing. The question of how the brain processes the imperfections of spontaneous speech is a key for shedding new light on this debate.

A word of caution when using multiple features in a regression analysis. As we have discussed, natural speech can be described on various levels, ranging from acoustic, linguistic, and semantic features to prosodic features. However, the selection of features for analysis needs to be considered carefully. Simply combining everything into a grand unifying model is ineffective. When performing TRF analysis for EEG/MEG data using multiple feature vectors, several key considerations must be taken into account to ensure accurate and meaningful results (Crosse et al., 2021). First, different feature vectors vary in the temporal resolution; for example, acoustic envelopes represent changes in the level of loudness at a fine-grained time scale, corresponding to the articulation time course (limited only by sample rate), whereas features of linguistic attributes represent information at varying time scales commensurate with the level of information to encode for (e.g., phonemes/words have inherently different temporal resolutions). Since, generally speaking, having more data or having data with high-density features leads to more reliable models, this could lead to differences in the reliability of models derived for different features.

Moreover, considering multiple feature vectors simultaneously increases model complexity. Models with too many feature vectors may overfit the data, while overly simple models may fail to capture or correct for important aspects of the neural response (Chen et al., 2023; de Heer et al., 2017; Mesik & Wojtczak, 2023). The choice of the time lag, that is, the range of delays between stimulus and neural response, is also critical. For low-level acoustic features, shorter lags are generally of interest compared to high-level features.

In summary, careful selection and consideration of features are essential for effective TRF analysis (Gillis et al., 2021; Mesik et al., 2021). By thoughtfully balancing feature complexity, temporal density, and data quantity, researchers can enhance the accuracy and meaningfulness of their TRF models.

### Language-Specific Challenges

Another point worth discussing is the availability and accuracy of automatic tools for speech annotation in different languages. The prevalence of English as a primary or secondary language and its dominance on the internet and in technology, has led to the fact that most automatic tools are developed first, and most extensively, in English (Martel, 2001; Wang et al., 2023). Both academic and commercial efforts devoted to developing automatic tools for language processing focus primarily on English, which has allowed for immense progress in the field. Despite increasing efforts to adapt tools from English to other languages, to date, these efforts have still been partial and vary greatly from language to language. This bias in the NLP world transfers to the field of neurolinguistics as well (Nee et al., 2022), since the access to appropriate tools dictates the type of research that can be conducted in neuroscience labs located in different countries, using participants’ mother tongue.

Advancing efforts to create language-specific models, and dedicating appropriate resources to this cause, is critical for enabling researchers worldwide to study how the brain processes language. This is, of course, an ambitious endeavor. It would not only require extensive corpora in each language, and taking note of dialectal variation and phoneme-to-grapheme mappings that are unique to each language, but would entail having these materials adequately transcribed and labeled for training purposes. Moreover, languages that use non-Latin orthography require additional efforts (as described in our pipeline for the case of Hebrew). Some of these challenges can be overcome through collaborative efforts and the sharing of annotated datasets as well as community-driven projects that can help build more extensive and accurate linguistic models for different languages. This collective approach will accelerate the development of NLP tools that are truly multilingual, enhancing the quality and inclusivity of speech annotation research.

### Data Sharing

Sharing annotated speech material in an accessible and reusable form is essential for ensuring replicability and facilitating reuse. Given the significant time and manual effort invested in annotating speech material, it is crucial to organize these resources in a format that is easily reusable by other researchers, maintaining the precise temporal connection between the sound file and its annotations. A widely used standard that supports the sharing of neuroimaging data is the Brain Imaging Data Structure (BIDS) framework (Gorgolewski et al., 2016). Complementing BIDS, Hierarchical Event Descriptors (HED; Robbins et al., 2021) have recently been introduced to describe stimulus material in a structured and machine-readable format. By adopting these standards, researchers can ensure their annotated speech materials are accessible and easily integrated into broader research efforts.

### Conclusion

This manuscript explores key issues regarding the tools necessary for studying the neural basis of processing everyday speech and introduces an annotation pipeline for this purpose. Our work demonstrates the potential of existing tools in deriving feature vectors while highlighting their limitations in faithfully capturing the intricacies of the acoustic signal, the very signal our brains rely on. We emphasize the significance of identifying suitable features for describing natural speech and elucidate why current methods often fail to capture these elements adequately.

To accurately annotate and analyze natural speech, we need to develop models that account for the unique characteristics of these spoken materials, treating the imperfect nature of speech as a feature, not a bug. This includes creating large, diverse datasets of speech transcriptions that capture all the nuances and complexities of natural speech, and training automatic tools on these materials, rather than relying on tools training on corpora of ideal texts. Moreover, it may be beneficial to create tools trained directly on audio data, rather than text, to better capture the dynamic and contextual aspects of spoken language.

We envision this manuscript not only as a methodological overview but as a call to action for the neurolinguistic community. To fully understand the neural basis of real-world language comprehension, we must embrace the complexity of natural speech—developing tools, datasets, and standards that reflect its inherently messy and dynamic nature. This includes investing in speech-trained NLP models, multilingual alignment tools, and community-driven efforts for annotated data sharing.

Through our work, we aim to inspire further research into addressing the challenges posed by natural speech comprehension. By refining feature extraction models and leveraging innovative methodologies, we can bridge existing gaps in transcription and analysis, ultimately advancing our understanding of how the brain processes spoken language. We encourage continued exploration and collaboration in this field to unlock the mysteries of natural speech and its underlying neural mechanisms.

## ACKNOWLEDGMENTS

We would like to thank Dr. Mareike Daeglau for providing access to the German speech material and Till Eric Wagner for his assistance in speech annotation. We would like to thank Evyatar Cohen, from the EasyAlignIPA development team at the Open University, for consulting on the development of the Hebrew pipeline. We gratefully acknowledge Dr. Amir Ivry (Electrical and Computer Engineering Faculty in The Technion) for his valuable advice and for sharing his expertise on audio-based models.

## FUNDING INFORMATION

Galit Agmon, U.S. Department of Defense (https://dx.doi.org/10.13039/100000005), Award ID: W81XWH-20-1-0531. Manuela Jaeger, Deutsche Forschungsgemeinschaft (https://dx.doi.org/10.13039/501100001659), Award ID: 490839860. Elana Zion Golumbic, Israel Science Foundation (https://dx.doi.org/10.13039/501100003977), Award ID: 2339/20. Elana Zion Golumbic, Deutsche Forschungsgemeinschaft (https://dx.doi.org/10.13039/501100001659), Award ID: 490839860. Martin G. Bleichner, Deutsche Forschungsgemeinschaft (https://dx.doi.org/10.13039/501100001659), Award ID: 490839860. Martin G. Bleichner, Deutsche Forschungsgemeinschaft (https://dx.doi.org/10.13039/501100001659), Award ID: 411333557.

## AUTHOR CONTRIBUTIONS

**Galit Agmon**: Conceptualization; Methodology; Writing – original draft; Writing – review & editing. **Manuela Jaeger**: Methodology; Writing – original draft; Writing – review & editing. **Ella Magen**: Methodology; Writing – original draft. **Danna Pinto**: Methodology; Writing – original draft; Writing – review & editing. **Yuval Perelmuter**: Methodology. **Elana Zion Golumbic**: Conceptualization; Funding acquisition; Methodology; Writing – original draft; Writing – review & editing. **Martin G. Bleichner**: Conceptualization; Funding acquisition; Methodology; Writing – original draft; Writing – review & editing.

## TECHNICAL TERMS

Temporal response function (TRF): Modeling approach that relates continuous stimulus features to time-resolved neural activity.
Large language models (LLMs): Neural network-based models trained to predict and generate human-like text; examples include GPT and BERT.
Verbatim transcription: An exact, word-for-word transcript of speech, including pauses, fillers, and self-corrections.
Forced alignment: Automatic matching of spoken audio with its transcript to assign time stamps to each word or phoneme.
Grapheme-to-phoneme conversion: Process of converting written text into its corresponding spoken sound units (phonemes).
